# Longitudinal Trends of Urologic Malignancy After Solid Organ Transplantation at a High-Volume Transplant Center

**DOI:** 10.7759/cureus.116771

**Published:** 2026-09-23

**Authors:** Gabrielle R Yankelevich, Luis G Medina, J. Peyton Vaughan, McKenzie Needham, Emma Bryant, Jennifer Drerup, Jean-Sebastien Anoma, Elizabeth Hill, Nicholas Zurinaga, Nikhil Anandpura, Vineeth Sama, Stephen J Savage

**Affiliations:** 1 Department of Urology, Medical University of South Carolina, Charleston, USA; 2 Department of Urology, Moffitt Cancer Center, Tampa, USA; 3 Department of Public Health Sciences, Medical University of South Carolina, Charleston, USA; 4 Department of Urology, Thomas Jefferson University, Philadelphia, USA

**Keywords:** cancer of kidney, post-transplant malignancy, prostate cancer, urology, urology and oncology, urothelial cell carcinoma

## Abstract

Introduction

Since the advent of solid organ transplantation, there have been many concerns related to the risks of immunosuppression and malignancy. The impact of transplantation on cancer risk warrants continued evaluation of contemporary malignancy patterns. We assessed current trends at our high-volume transplant center.

Methods

A single-center retrospective analysis was performed at a high-volume transplant center in South Carolina for adult patients with a history of solid organ transplant who subsequently had a new urologic malignancy between 2008 and 2023. The malignancies were delineated with International Classification of Diseases, 10th Revision (ICD-10) codes for the bladder, urethra, ureter, renal pelvis, kidney, prostate, adrenal, penis, and testis. Demographic, transplant, and malignancy data were analyzed. Outcomes were analyzed for mortality rate, and univariate survival analysis was performed.

Results

Eighty-one patients met the inclusion criteria. The mean age at transplant was 56 years, and the mean age at cancer diagnosis was 60 years. The breakdown of cancers from most to least common was prostate (41%, 33), kidney (38%, 31), bladder (19%, 15), upper tract urothelial (1%, 1), and urethra (1%, 1). The mortality rate was 0.06 for urothelial, 0.05 for kidney, and 0.02 for prostate, compared with national averages of 0.005, 0.005, and 0.011, respectively. Survival analysis demonstrated significantly decreased survival for patients with urothelial malignancy after transplant compared with prostate cancer (p<0.01).

Conclusions

We found a significantly increased proportion of kidney and bladder malignancy in transplant patients in comparison to the South Carolina and national yearly incidence of these cancers in our single-institution high-volume transplant center.

## Introduction

Solid organ transplants are increasing, with 2024 national data showing 48,149 transplants performed, 3.3% more than 2023 and 23.3% more than the prior five years [1]. Data collected by the World Health Organization-Global Observatory on Donation and Transplantation from 2008 to 2023 demonstrated that the number of transplants performed worldwide has increased by 76% during this period [2]. Of the transplants performed, kidney transplants represent the majority, representing 64% of transplants performed in 2023 worldwide [2].

While cardiovascular disease is the leading cause of death after transplant, malignancy accounts for the second most common cause of death, with a 10%-15% incidence of solid organ cancer at 15 years post-transplant [3]. Several studies have demonstrated that among renal transplant recipients, urologic malignancies have a higher incidence in comparison to population-matched controls, with renal cell cancer (RCC) being the most common urologic cancer [3-5]. The incidence of RCC has been reported to be between 0.8 and 2.1, with higher incidences of papillary type RCC when compared with the general public [4-8]. Similar to RCC, there is an associated increased risk of urothelial carcinoma (UCC) in kidney transplant recipients compared with the general public, with several studies reporting polyomaviruses as a plausible explanation for the increased risk [9-11]. A prior study reports the risk of RCC and UCC after kidney transplant to be sevenfold and threefold, respectively [12]. However, there is a lack of contemporary data despite the increase in transplants, modifications in immunosuppression, and advances in cancer diagnostics. Therefore, we evaluated the current incidence and survival outcomes of urologic malignancies in the transplant population at a high-volume transplant center in South Carolina (SC).

## Materials and methods

After Institutional Review Board approval, we conducted a retrospective cohort study of patients who underwent a transplant procedure between 2008 and 2023 and subsequently developed a new urologic malignancy diagnosis. Inclusion criteria were age ≥18 years, transplant performed at a single high-volume transplant center, and diagnosis of malignancy after transplant. Exclusion criteria were transplants performed outside of our transplant center and urologic malignancy diagnosed before transplant. Clinical and demographic characteristics, including age, sex, race, type of transplant (kidney, kidney-pancreas, liver, liver-kidney, heart-kidney, heart, and lung), date of transplant, malignancy type (bladder, urethra, ureter, renal pelvis, kidney, prostate, adrenal, penis, and testis), and date of malignancy diagnosis were collected.

Urologic malignancies included were urothelial (bladder, urethra, upper tract urothelial), kidney, prostate, adrenal, penis, and testis, categorized by International Classification of Diseases, 10th Revision (ICD-10) codes. Descriptive statistics summarized clinical and demographic characteristics, with categorical variables reported as frequencies and percentages and continuous variables as means with standard deviations (SDs) or medians with interquartile ranges, as appropriate. Categorical outcomes included the proportion of urologic malignancies and the mortality rate in the transplant population. The SC incidence rates were obtained from the state cancer database and national data from the Surveillance, Epidemiology, and End Results (SEER) database [13,14]. Survival outcomes were evaluated using the Kaplan-Meier method, and differences in overall survival between groups (prostate vs. urothelial or kidney) were assessed using the log-rank test. Univariate Cox proportional hazards regression models were constructed for each group of interest to estimate associations with survival outcomes, and hazard ratios with 95% confidence intervals were reported. Statistical significance was defined as a two-sided p-value <0.05, and analyses were performed in R version 4.3.0 (R Foundation for Statistical Computing, Vienna, Austria) using the following packages: “gtsummary” (version 2.4.0), “survival” (version 3.8-3), “survminer” (version 0.5.1).

## Results

A total of 81 patients met the inclusion criteria out of 5,869 transplants performed. The mean age at transplant was 56 years (SD: 11.6), and the mean age at cancer diagnosis was 60 years (SD: 11.0). The demographics are shown in Table 1. The most common malignancy was prostate cancer (41%, 33), followed closely by kidney cancer (38%, 31) and bladder cancer (19%, 15). There was only one case of upper tract UCC (comprised of ureter and renal pelvis, UTUC) and one case of urethral malignancy. These findings are shown in Table 2. When stratifying by heart and lung transplants, similar trends were seen, with the most common being prostate cancer (59%, 10), followed by bladder malignancy (29%, 5) and renal carcinoma (12%, 2). 

**Table 1:** Baseline demographics and clinical characteristics of transplant population

Demographic	n	%
Sex		
Male	70	86.4
Female	11	13.6
Race		
White	33	40.7
Black/African American	48	59.3
Body mass index	28.6	35.3
Smoking history		
Yes	45	55.6
No	36	44.4
Alcohol use		
Yes	30	37
No	51	63
Coronary artery disease or congestive heart failure		
Yes	37	45.7
No	44	54.3
Hypertension		
Yes	72	88.9
No	9	11.1
Hyperlipidemia		
Yes	57	70.4
No	24	29.6
Chronic obstructive pulmonary disease		
Yes	9	11.1
No	72	88.9
Diabetes mellitus type 2		
Yes	41	50.6
No	40	49.4
Other prior malignancy		
Yes	15	18.5
No	66	81.5

**Table 2:** Proportion of urologic malignancy in transplant population

Malignancy	Proportion of cancer, % (n)
Prostate	41% (33)
Kidney	38% (31)
Bladder	19% (15)
Upper tract urothelial	1% (1)
Urethra	1% (1)

The incidence rate was calculated and compared with SC and national incidence rates, as shown in Table 3. Bladder malignancy incidence was about 10-fold higher, at 0.17 per 1000 people-years, than SC and national rates of 0.017 and 0.025 per 1000 people-years. The kidney cancer incidence was 0.35 people-years, significantly higher than SC and national incidence rates of 0.018 and 0.025 people-years. The prostate cancer incidence was 0.38 people-years, compared with SC and national incidence rates of 0.115 and 0.092 per 1000 people-years.

**Table 3:** Incidence rate in transplant population compared with SC incidence, national incidence, and lifetime risk SC, South Carolina.

Malignancy	Incidence rate in transplant population	SC incidence rate	National incidence rate	National lifetime risk
Bladder	0.17	0.017	0.025	Men 3.57%, women 1.1%
Kidney	0.35	0.018	0.024	Men 2.3%, women 1.4%
Prostate	0.38	0.115	0.092	Men 12.5%

The mortality rate was calculated and compared to the national mortality rate, as shown (Table 4). The national data were obtained from the SEER database. Given that there was only one case of UTUC and one case of urethral malignancy, both with urothelial pathology, these were combined with bladder to make a urothelial group when assessing mortality. Moreover, the mortality rates are reported for UCC as a grouped disease rather than individual organs. The mortality rate in the transplant population was 0.06, over 10 times higher than the national mortality rate for UCC at 0.005. Likewise, the mortality rate for kidney cancer was 0.05, over ten-fold higher than the national mortality rate of 0.004. The prostate cancer mortality rate was 0.02, compared with a national mortality rate of 0.011.

**Table 4:** Mortality rate for urologic malignancy in transplant population

Malignancy	Mortality rate in transplant population	National mortality rate
Urothelial	0.06	0.005
Kidney	0.05	0.004
Prostate	0.02	0.011

Survival analysis was performed and compared urothelial cancer with prostate cancer, showing significantly decreased survival for patients with urothelial malignancy (p=0.01), as shown (Figure 1). Univariate analysis showed that urothelial disease was significantly associated with death from disease (p=0.04), with no correlation to smoking status, age, race, and BMI.

**Figure 1:**
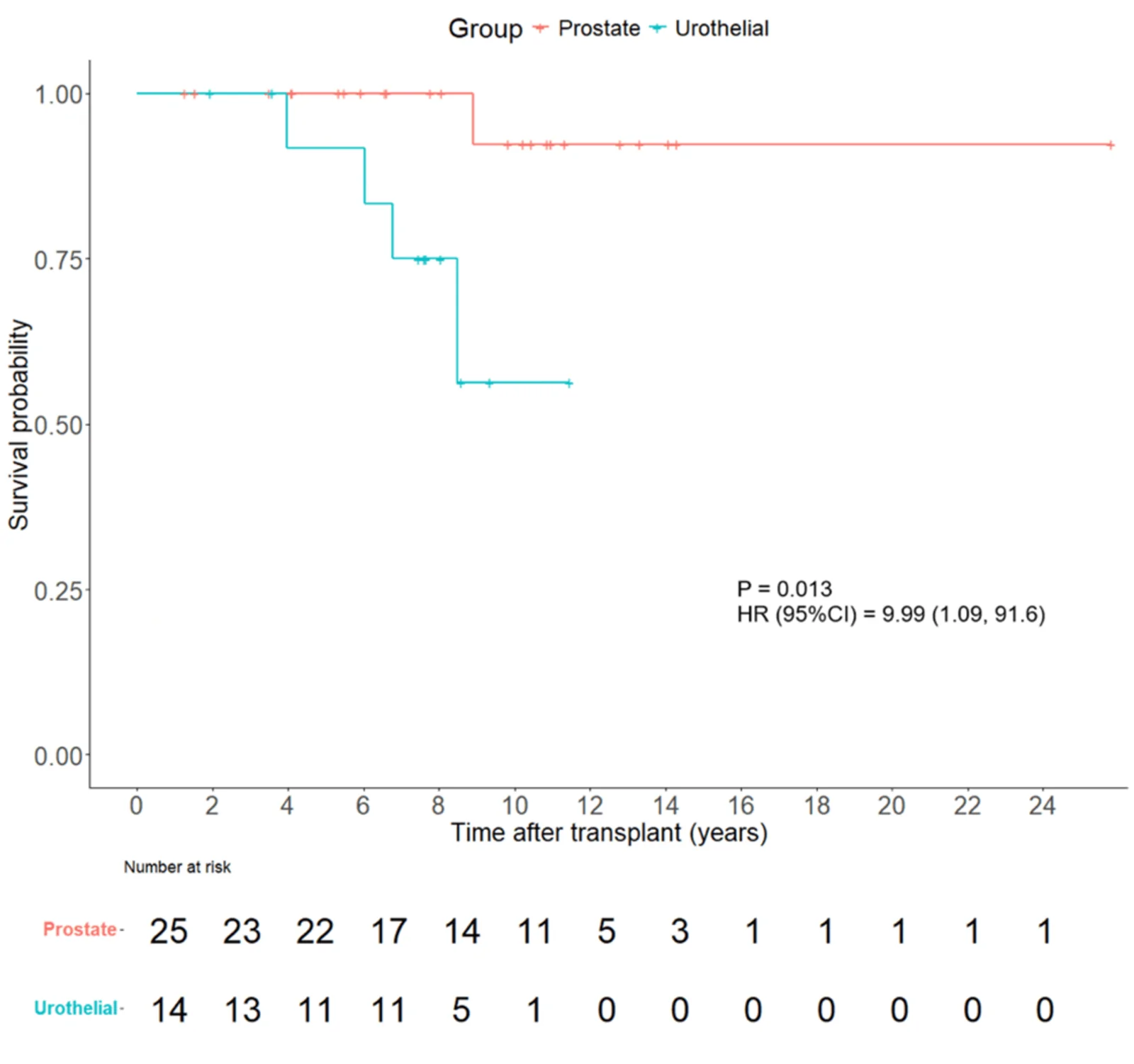
Kaplan-Meier curve for urothelial cancer compared with prostate cancer

## Discussion

In this study period, we found that 1.4% of transplant patients developed a urologic malignancy. The most common malignancy was prostate cancer, followed by kidney cancer and then UCC. There are limited earlier reports that assess malignancy risk among all solid organ transplants, as the vast majority of the literature addresses this in the context of prior kidney transplant. In 2010, Di Capua Sacoto et al. performed a descriptive analysis of de novo urologic tumors in kidney transplant patients, finding 1.6% with a urologic malignancy [15]. Similar to our study, where the tumors were diagnosed at a median of four years after transplant, they found the tumors were diagnosed at a median of 5.6 years after transplant [15]. Antunes et al. conducted a retrospective study in kidney transplant recipients, similarly showing 2.2% with a urologic cancer. Similar to our study, the majority of patients were affected with prostate cancer; however, there was one reported case of penile carcinoma [16]. Lastly, Lu and colleagues performed a similar study in renal transplant patients, but due to their long follow-up period, found a bimodal trend in the risk of occurrence of urologic malignancies. This trend remained true when stratifying for age and gender [16].

When looking at transplant recipients, several risk factors have been proposed as to why the malignancy risk is increased. Immunosuppression is a necessity to prevent rejection, usually in the form of calcineurin inhibitors, antimetabolites, and steroids [17-19]. However, these treatments impact T-cell-mediated immunosurveillance, which allows for normal detection and elimination of transformed cells, which can therefore lead to malignant cells [3,10,16-20]. In the kidney transplant population, immunosuppression can lead to viral infections such as human polyomavirus, most commonly BK virus [12,18]. BK virus leads to malignancy, as it can bind to and inactivate a host’s tumor suppressor genes [18]. Likewise, Epstein-Barr virus, human T-cell lymphotropic virus type 1, Kaposi’s sarcoma-associated herpes virus, and hepatitis viruses are known oncogenic viruses that lead to non-urologic malignancies [3,17,19].

When looking at risk specifically for each organ, some proposed pathways for malignancy have also been suggested. For prostate cancer, some studies have found that sirolimus is associated with increased prostate cancer incidence; however, others have shown no increased risk of prostate cancer compared with the general population [12,17]. Risk factors for renal carcinoma include abuse of painkillers, acquired cysts in native kidneys, older age, male gender, smoking, and longer time on dialysis (more than three years) [3,12,17,20]. Specific to those who were on dialysis before transplant, an increased risk of urothelial cancer has been seen, with longer duration of dialysis before transplant increasing the risk by 2.5 times [17,18]. This has been attributed to end-stage renal disease, as decreased urine output leads to retention of metabolic toxins such as aromatic amines and polycyclic aromatic hydrocarbons, which can provoke DNA damage in urothelial cells due to chronic oxidative stress [18]. Likewise, BK virus has been identified as a risk factor for urothelial cancer, with the presence in kidney transplant recipients being associated with a fivefold increase in UCC compared with those without BK virus [12].

Lastly, the reasoning for increased rates of urothelial and kidney cancer over other urologic malignancies could be attributed to the immunology of the cancer itself. Urothelial and kidney cancer have been noted to be immunomodulated, whereas prostate, adrenal, penile, and testicular cancer are non-immunomodulated. UCC is known to be sensitive to immunotherapy in non-muscle invasive disease, muscle invasive disease, and metastatic disease, which is why intravesical immunotherapy such as Bacillus Calmette-Guérin or systemic immunotherapy is exceedingly common for treatment [21]. Similarly, renal malignancy has been known to be immunomodulated, as initial treatments targeted interleukin-2 and interferon alpha, though this has progressed to using checkpoint inhibitors such as nivolumab or vascular endothelial growth factor inhibitors such as bevacizumab [21]. Within prostate cancer, some studies for metastatic disease support the use of immune checkpoint inhibitors. However, other studies have reported that immunotherapy has limited clinical efficacy given that prostate cancer has been identified as a “cold tumor” [22]. Though data are emerging for the treatment of adrenal, penile, and testicular cancer with immunotherapy, these treatments are typically reserved for patients who fail to achieve cure with chemotherapy or have aggressive and advanced disease [21,23,24].

The incidence rate of bladder cancer (0.17 per 1000 people-years) was about 10-fold higher than the state (SC) and national incidence rates (0.017 and 0.025), and likewise the mortality of UCC (0.06 per 1000 people-years) was over 10 times higher than the national mortality rate (0.005). For kidney cancer, the incidence (0.35 people-years) and mortality rate (0.05 per 1000 people-years) were significantly higher than national rates (0.025 and 0.004). Lastly, prostate cancer was the most common but had the lowest mortality rate at 0.02 per 1000 people-years compared with a national mortality rate of 0.011. A similar study performed by Ye et al. mirrored ours, with bladder cancer having the worst five-year survival, followed by renal cancer and then prostate cancer [25].

The overall risk of cancer death after transplant is increased in comparison to an age- and sex-matched population due to being more aggressive and having a higher stage [26]. One explanation for why cancers are associated with worse outcomes in transplant patients proposed is that tumors that arise in an immunosuppressed setting are more aggressive [26]. Moreover, once a patient has cancer, the immunosuppression drugs promote angiogenesis and potentially allow the tumors to invade into surrounding tissues [27].

The elevated incidence and mortality rates suggest the potential need for increased vigilance in screening practices for urologic malignancies in transplant patients, particularly for UCC, which is associated with poorer survival outcomes. The American Society of Transplantation recommends screening in solid organ transplant recipients for skin, breast, cervical, colorectal, prostate, and liver cancer; however, guidelines do not exist on screening for urothelial malignancy [28]. Likewise, screening for kidney cancer is not recommended; however, it is noted that European Best Practice Guidelines recommend ultrasound screening of native kidneys among kidney transplant patients [28]. Some studies have recommended ultrasounds and urinalysis, but there is sparse data on this [18,29]. Therefore, we propose annual ultrasound and urinalysis, as these are inexpensive tests that can identify urothelial or renal cell carcinomas in a high-risk population. Newer screening modalities may be beneficial in the future, such as genomics and molecular markers [30].

In our study, there remains a knowledge gap for adrenal, testicular, and penile cancer, as there were no patients with these malignancies in our cohort. While the incidence of these urologic malignancies increased in the post-transplant population, it is unclear whether incidence is impacted due to this being a closely followed population.

Limitations of the study include the retrospective design and the need to compare the retrospective cohort with the SEER database. The exact mechanism of diagnosis and adherence to screening guidelines is unknown. Despite being retrospective, the number of transplants was high, though the number of events per specific malignancy was low; therefore, the results should not be taken as definitive, and future prospective studies could be evaluated. Next, given significant heterogeneity in transplant immunosuppression medications across all transplants, we are unable to comment on the effect of immunosuppression drugs on the development of malignancy. Lastly, although this was performed at a high-volume transplant center, the results are from a single institution and may therefore limit generalizability. 

## Conclusions

Ultimately, this study found a significantly increased proportion of urothelial, kidney, and prostate malignancies in post-transplant patients in comparison to the incidence rate in the general population and in SC at our single-institution high-volume transplant center. There was significantly worse survival for those who had UCC after transplant. A better understanding of the molecular mechanisms may allow improved tailored diagnosis and treatment.
